# Enhancement of Cellulase Production in *Trichoderma reesei* via Disruption of Multiple Protease Genes Identified by Comparative Secretomics

**DOI:** 10.3389/fmicb.2019.02784

**Published:** 2019-12-03

**Authors:** Yuanchao Qian, Lixia Zhong, Yu Sun, Ningning Sun, Lei Zhang, Weifeng Liu, Yinbo Qu, Yaohua Zhong

**Affiliations:** ^1^State Key Laboratory of Microbial Technology, Institute of Microbial Technology, Shandong University, Qingdao, China; ^2^Shandong Institute for Food and Drug Control, Jinan, China; ^3^Department of Immunology, School of Basic Medical Sciences, Shandong University, Jinan, China

**Keywords:** *Trichoderma reesei*, protease, cellulase, comparative secretomics, gene deletion

## Abstract

The filamentous fungus *Trichoderma reesei* is one of the most studied cellulolytic organisms and the major producer of cellulases for industrial applications. However, undesired degradation of cellulases often happens in culture filtrates and commercial enzyme preparations. Even studies have been reported about describing proteolytic degradation of heterologous proteins in *T. reesei*, there are few systematic explorations concerning the extracellular proteases responsible for degradation of cellulases. In this study, the cellulase activity was observed to rapidly decrease at late cultivation stages using corn steep liquor (CSL) as the nitrogen source in *T. reesei*. It was discovered that this decrease may be caused by proteases. To identify the proteases, comparative secretomics was performed to analyze the concomitant proteases during the cellulase production. 12 candidate proteases from the secretome of *T. reesei* were identified and their encoding genes were individually deleted via homologous recombination. Furthermore, three target proteases (tre81070, tre120998, and tre123234) were simultaneously deleted by one-step genetic transformation. The triple deletion strain ΔP70 showed a 78% decrease in protease activity and a six-fold increase in cellulase activity at late fermentation stages. These results demonstrated the feasibility of improvement of cellulase production by genetically disrupting the potential protease genes to construct the *T. reesei* strains with low extracellular protease secretion. This dataset also provides an efficient approach for strain improvement by precise genetic engineering combined with “omics” strategy for high-production of industrial enzymes to reduce the cost of lignocellulose bioconversion.

## Introduction

The filamentous fungus *Trichoderma reesei* is one of the most investigated cellulolytic organisms for the robust capacity to produce the large amounts of lignocellulolytic enzymes (Cherry and Fidantsef, 2003). This complex enzyme system consists of cellulases, such as cellobiohydrolase (CBH) and endo-β-1,4-glucanase (EG), and hemicellulases, such as xylanase (XYN), which cooperate to complete biomass hydrolysis (Singhania et al., 2013). In addition, the production of lignocellulolytic enzymes could achieve the level of 100 g/L (Cherry and Fidantsef, 2003). So up to now, it is the dominant producer for production of the current commercial cellulase preparation (Singhania et al., 2013; Landowski et al., 2015). However, the truncated cellulases had been detected in the culture filtrates and the commercial enzyme preparations (Kubicek-Pranz et al., 1991; Collén et al., 2011). It was further demonstrated that the occurrence of truncated cellulases were probably due to their hydrolysis by proteases (Dienes et al., 2007). Therefore, the formation of extracellular proteases of *T. reesei* is a limitation for the production of cellulase during the fermentation process. To overcome this problem, low-protease-level strains or protease-deficient strains should be developed or isolated to improve cellulase production.

Both random mutagenesis and genetic modification are the frequently used strategies for generating the fungal strains with increased or reduced levels of targeted products in filamentous fungi (Punt et al., 2008). Apparently, the random mutagenesis approach results in the potent production hosts, but the genetic bases of these mutants remain unknown and may have undesired effects on fungal fermentation performance, such as low growth rate (Peterson and Nevalainen, 2012). Compared with random mutagenesis, genetic modification provides a more rational approach to obtain the targeted strains. Actually, genetic modification has been applied to generate strains with reduced protease activity for increasing the protein production by *Aspergillus* sp. (Punt et al., 2008; Yoon et al., 2009; Sriranganadane et al., 2011). Recently, several studies with the genetic modification method have been carried out to generate the low-protease-level strains in *T. reesei* (Zhang et al., 2014; Landowski et al., 2016). The production of the heterologous alkaline EG was improved in the alkaline protease-disrupted strain *T. reesei* Δ*spw1* (Zhang et al., 2014). Deletion of the subtilisin protease gene *slp7* or the metalloprotease gene *amp2* in *T. reesei* has enhanced the production level of heterologous IFNα-2b (Landowski et al., 2016). Evidently, it is feasible to construct the protease deficient strains via the genetic manipulation for improving the production of extracellular protein in *T. reesei.* However, considering the heterologous proteins often subjected to degradation easier than homologous proteins (Zhang et al., 2014), these results may be not applied to the endogenous cellulase production in *T. reesei.* Indeed, two other acid proteases were identified to play a partial role in degradation of the homologous cellulase (Dienes et al., 2007). Therefore, more investigations are required to study the protease secretion profile during fermentation process for cellulase production in *T. reesei*. Such investigations are necessary to construct the protease-deficient strains for high-level production of cellulases.

Approximately, 200 genes involved in proteolytic degradation are present in the genome of *T. reesei* (Martinez et al., 2008). Considering the high number of putative proteases, it may be impractical to delete them all (Landowski et al., 2015). It is known that the fungal extracellular proteases could degrade protein into oligopeptides or free amino acids to provide the nitrogen source for fungal growth, particularly when nitrogen is limited. Thus, it seems unrealistic and inadvisable to construct the strains with no extracellular protease activities (Yoon et al., 2009). Therefore, identification of the specific proteases associated with cellulase degradation should be the prerequisite to construct the protease-deficient strains. Proteomics provides an excellent tool for discovering and identifying the secreted proteins under variable conditions (Adav et al., 2010, 2011). Particularly, the liquid chromatography-tandem mass spectrometry (LC-MS/MS) has been shown to be one of the most efficient proteomics strategies for identification of thousands of proteins in a complex sample (Gong et al., 2015; Byrum et al., 2018). In this study, the LC-MS/MS method was employed to identify the extracellular proteases associated with cellulase degradation during fermentation process. The candidate protease genes were successively deleted and the low-protease-level *T. reesei* strain was finally constructed by knocking out the target protease genes with one-step genetic manipulation. This strain with the multiple protease deletions possessed the stable expression levels of endogenous cellulases and thus a more productive *T. reesei* strain suitable for cellulase production.

## Materials and Methods

### Strains and Culture Conditions

*Trichoderma reesei* QM9414Δ*mus53*, a hyper-cellulolytic strain with high-efficiency homologous recombination, was kindly provided by Häkkinen et al. (2014) and used as the host strain for gene deletion. All strains were cultured on potato dextrose agar (PDA) plates until generating spores. Equal concentrations of conidia (10^6^ spores per mL) were inoculated into 50 mL of seed medium in 500 mL Erlenmeyer flasks and cultured at 200 rpm and 30°C for 36 h. Then the 10 mL of pre-cultures were transferred into 100 mL of the cellulase production medium (CPM) in 500 mL Erlenmeyer flasks and were incubated at 200 rpm and 30°C. The fermentation supernatants were taken every 24 h for cellulase production assay. The composition of seed medium was as follows (g/L): 10 glucose, 5 (NH_4_)_2_SO_4_, 15 KH_2_PO_4_, 0.6 CaCl_2_.2H_2_O, 0.5 MgSO_4_.7H_2_O, and 2 peptone. The composition of CPM was as follows (g/L): 20 microcrystalline cellulose, 15 KH_2_PO_4_, 0.6 CaCl_2_.2H_2_O, 0.5 MgSO_4_.7H_2_O, and 5 (NH_4_)_2_SO_4_ or 20 corn steep liquor (CSL).

### Gelatin Zymography

Gelatin zymography of protease activity was performed mainly by the previously described method with modification (Landowski et al., 2015). Briefly, crude enzymes were separated on 12% SDS-PAGE with 0.1% gelatin (Dingguo Corp., Beijing, China). After running the gel, proteins in the gel were renatured two times for 30 min in 2.5% Triton X-100 and 50 mmol/L Tris–HCL and then washed by 50 mmol/L Tris–HCL to further remove the SDS. The zymogram gel was then allowed to incubate overnight in reaction buffer (50 mM sodium citrate, pH 5.5). Then, the gel was stained with Coomassie Blue (Thermo Scientific) and destined in 10% acetic acid/ethanol.

### Liquid Chromatography-Tandem Mass Spectrometry (LC-MS/MS)

Liquid chromatography-tandem mass spectrometry was performed to identify the extracellular proteases according to the method previously described with some modifications (Song et al., 2016). The fermentation supernatants were centrifuged and then filtered through 0.22 μm PES membrane (Millipore) to remove the mycelium. The proteins in the supernatant were precipitated by acetone and trichloroacetic acid. The resuspended precipitates were dissolved in ultrapure water. And then 100 μg (>10 mg/mL) of proteins were degenerated by 50 μL of degeneration buffer (0.5 M Tris–HCl, 2.75 mM ethylenediaminetetraacetic acid (EDTA), 6 M guanidine-HCl, pH 8.1) and reduced by 50 μL of 1 M dithiothreitol (DTT) at 37°C for 2 h. Then, the proteins were alkylated by 50 μL of 1 M iodoacetamide for 1 h under the dark condition. The alkylated proteins were filtered and collected by the Microcon YM-10 centrifugal filter (Millipore Corporation, United States). After centrifugation, the proteins were digested by trypsin (Sigma, United States) at 37°C for overnight. The digested oligopeptides were desalted by the ZipTip C18 column (Millipore Corporation, United States) and were further dissolved in 10 μL of elution buffer [50% (v/v) acetonitrile (ACN) and 0.1% (v/v) trifluoroacetic acid] and then were subjected to nanoelectrospray ionization, followed by tandem mass spectrometry (MS/MS) in an LTQ Orbitrap Velos Pro (Thermo Scientific^TM^, United States) coupled inline to HPLC. The peptides were detected by the Orbitrap at a resolution of 60,000. Data resulting from LC-MS/MS analysis of trypsin digested proteins were searched by proteome discovered software 1.4 (Thermo Fisher Scientific) with the SEQUEST using the *T. reesei* database^1^. Functional matching of identified proteins was conducted using SEQUEST. The MEROPS database was adopted for protease identification^2^.

### Gene Deletion

To construct the protease gene-deletion strains, the gene knocked-out cassettes of 12 protease genes: *tre22459*, *tre51365*, *tre77579, tre73897*, *tre77579*, *tre79809*, *tre81070*, *tre81517*, *tre103039*, *tre105808*, *tre120998*, *tre123234*, and *tre123244* were constructed using the double-joint PCR method as previously described (Yu et al., 2004). These cassettes containing the selectable marker *hph* flanked by the 5′- and 3′-flanking regions of the corresponding protease genes (Supplementary Figure S3a) were transformed into the protoplasts of *T. reesei* with the PEG-mediated transformation method as described previously (Penttilä et al., 1987). For construction of the triple-deletion strain, three gene knocked-out cassettes for *tre81070*, *tre120998*, and *tre123234* at the same molar concentration were simultaneously transformed into the protoplasts QM9414Δ*mus53.* The transformants were directly screened on MM plates containing the 300 μg/mL hygromycin B. The purified candidates were further performed though PCR or Southern blot analysis to verify if the corresponding protease genes locus were replaced by the knocked-out cassette. The primers used in construction and verification of the corresponding gene deletions were listed in Supplementary Table S1.

### Southern Blot Analysis

Transformants were subjected to Southern blot analysis to dissect the integration types of the gene knocked-out cassettes. The probes of the corresponding genes were amplified through PCR by using the probe primers (Supplementary Table S1). In detail, the primer pairs Probe-81070-F/Probe-81070-R, Probe-120998-F/Probe-120998-R, and Probe-123234-F/Probe-123234-R were used to prepare for the probes of *tre81070*, *tre120998*, and *tre123234*, respectively. The genomic DNA was digested by the restriction enzyme *Xho*I, separated on a 0.8% (wt/vol) agarose gel and then transferred to a nylon membrane (Hybond N+, Amersham). Probe labeling, hybridization and detection were carried out according to the manufacturer’s recommendations for the use of the DIG High Primer DNA Labeling and Detection Starter Kit I (Roche Applied Science, Mannheim, Germany).

### Fungal Growth

To analyze the effect of different carbon sources or nitrogen sources on growth, equal concentrations of spores (10^6^ spores per mL) were inoculated on the central of agar plates supplemented with 0.2% peptone used as nitrogen sources and 2.0% (w/v) of the different carbon sources (glucose, lactose or glycerol) for 4 days at 30°C. To evaluate the influence of the protease gene deletion on nitrogen utilization, equivalent amount of spores were inoculated on the central of agar plates that contained 2.0% glucose as carbon source and 2.0% (w/v) of the casein, gelatin, milk or peptone the nitrogen sources at 30°C for 4 days. The colony diameters of the protease gene deletion strains and the parental strain QM9414Δ*mus53* were measured at 24, 44, 56, 68, and 76 h after incubation.

### Determination of Cellulase Activity, Protease Activity, and Protein Concentration

The activities of Filter paper (total cellulase activity), EGs and xylanases were measured using Whatman No. 1 filter paper, CMC-Na and xylan as substrates, respectively. Then reaction mixtures contained 50 mg of filter paper, 1.0% CMC-Na or 1.0% xylan with 500μL of the suitably diluted enzyme fractions. These mixtures were then incubated at 50°C for 60 min (FPA) or 30 min (EGs and xylanases activities). The amount of reducing sugar released was determined using the DNS method (Ghose, 1987).

The activities of CBHs and β-glucosidase (BGL) were determined according to Ghose with modifications using p-nitrophenyl-β-D-cellobioside (pNPC) and p-nitrophenyl-β-D-glucopyranoside (pNPG) as substrates, respectively (Ghose, 1987). The diluted supernatants (100 μL) were incubated with 50 μL of 10 mM pNPG or 10 mg/mL pNPG dissolved in 50 mM acetate buffer (pH 5.0) at 50°C for 30 min. Then, 150 μL of each sample was mixed with an equal volume of 10% sodium carbonate. The absorbance at 420 nm was measured. One unit of EGs or BGL activity was defined as the amount of enzyme releasing 1 μmol of pNP per minute.

Protease activity was measured as described previously with modifications (Lovrien et al., 1985). Briefly, the 100 μL diluted supernatants were incubated with 400 μL of 0.25% (w/v) azocasein dissolved in 100 mM phosphoric buffer (pH 7.2) at 37°C for 2 h. Then, the reaction was terminated by the addition of 400 μL of 10% trichloroacetic acid and then centrifuged at 10,000 rpm for 10 min. After centrifugation, 100 μL of the supernatants were collected and mixed with 500 μL of 10% Na_2_CO_3_ to terminate the reactions and the absorbance was measured at 440 nm. Control assay without enzyme was used as the blank. Total extracellular protein concentration was measured with the Bio-Rad Protein Assay Kit (Bio-Rad, United States) following the manufacturer’s instructions.

## Results and Discussion

### Production of Extracellular Protein Decreased Significantly at Late Cultivation Stages in *T. reesei*

**FIGURE 1:**
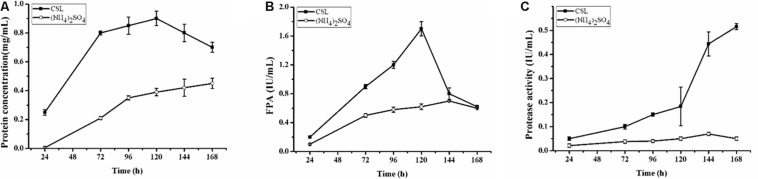
Cellulase and protease production by *T. reesei* during cultivation in CPM containing different nitrogen sources: 0.5% (w/v) (NH4)_2_SO_4_ (open cycle) or 2.0% corn steep liquor (CSL, filled square). **(A)** The extracellular protein produced by *T. reesei* was examined. **(B)** The total cellulase activities (FPA) were assayed. **(C)** Time course analysis of the protease activity levels during cultivation in the CPM media containing different nitrogen sources: 0.5% (w/v) (NH4)_2_SO_4_ (open cycle) or 2.0% corn steep liquor (CSL, filled square). Data are the means of three independent experiments; error bars show standard deviations.

The complex nitrogen sources, such as CSL and yeast extract, have been regarded as the popular organic nitrogen sources to improve the extracellular protein yields in filamentous fungi (Facciotti et al., 1991; Farid and El-Shahed, 1993; Pedersen and Nielsen, 2000). Especially, CSL is the preferred substrate for cellulase production due to its nutritional richness in organic nitrogen sources to promote fungal growth (Rana et al., 2014). To evaluate the effect of CSL on cellulase production, CSL was used as the nitrogen source to cultivate the hyper-cellulolytic strain QM9414Δ*mus53*. As shown in Figure 1A, the concentration of extracellular protein reached 0.90 mg/mL with CSL as the nitrogen source, which was much higher than that with (NH_4_)_2_SO_4_ (0.39 mg/mL). Correspondingly, the highest cellulase activity (1.70 IU/mL) under CSL condition was obtained at 120 h of cultivation, which was also significantly higher than that in (NH_4_)_2_SO_4_ (0.62 IU/mL) (Figure 1B). However, the cellulase activity under CSL condition was rapidly decreased at late cultivation stages after 120 h, where the cellulase production was reduced by 64% at 168 h in comparison to that at 120 h (Figure 1B).

**FIGURE 2:**
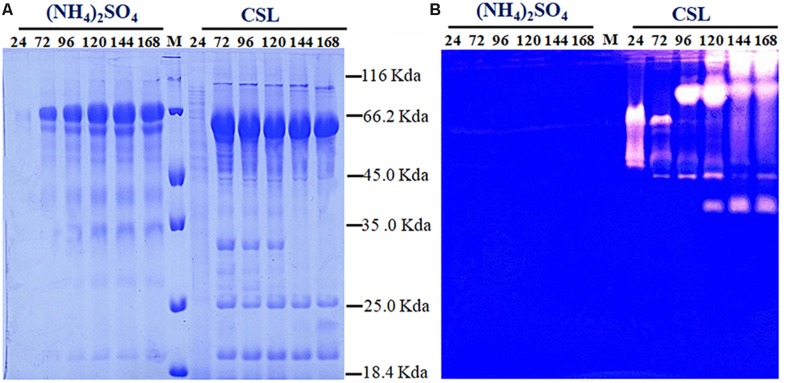
Determination of the extracellular protein and protease activity of *T. reesei*. **(A)** SDS-PAGE analysis of the supernatants from *T. reesei.* **(B)** Gelatin zymography analysis of the proteases at different cultivation stages, including 24, 72, 96, 120, 144, and 168 h, in the CPM media containing (NH_4_)_2_SO_4_ or corn steep liquor (CSL). Data are the means of three independent experiments; error bars show standard deviations.

Proteolytic degradation of cellulases and other extracellular proteins produced by *T. reesei* has been attributed to proteases (Eneyskaya et al., 1999). And a trypsin-like serine protease was identified as the major factor for Cel7B truncation in *T. reesei* (Dienes et al., 2007). Thus, the possible reason why a rapid decrease of cellulase activity occurred during the CSL cultivation in this study might be the extracellular proteases induced by CSL but not by (NH_4_)_2_SO_4_. To test this hypothesis, the protease activities of the culture supernatants of *T. reesei* under conditions of CSL and (NH_4_)_2_SO_4_ were measured. It was found that the protease activity increased significantly at 120 h in CSL while the protease activity was not changed in (NH_4_)_2_SO_4_ (Figure 1C). Furthermore, the culture supernatants were assayed by SDS-PAGE and gelatin zymography. As shown in Figure 2A, the amount of protein significantly decreased after 120 h under CSL condition, while there was no decrease under (NH_4_)_2_SO_4_ condition. As shown in Figure 2B, a small amount of proteases with relatively low molecular weight (30–50 kDa) were present before 120 h and much more proteases with relatively high molecular weight (60–200 kDa) occurred after 120 h under CSL condition, while there was no band present under (NH_4_)_2_SO_4_ condition. As for the reason for enhancement of protease activity, it was probably due to the lack of available preferred nitrogen sources in the media at the late cultivation stages and thus inducing the protease production. Taken together, the results suggested that there was a close correlation between the occurrence of extracellular proteases and the degradation of cellulases under CSL condition at late fermentation stages.

### Identification of the Proteases in the Secretome of *T. reesei*

**FIGURE 3:**
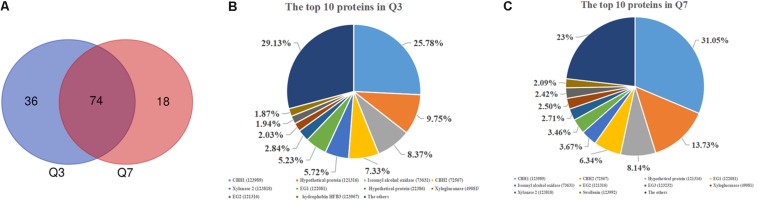
The secreted proteins in the 72 h (Q3) and 168 h (Q7) fermentation supernatants of *T. reesei* identified by means of LC-MS/MS. **(A)** Venn diagram representing the number of secreted proteins by the *T. reesei* at 72 h (Q3) and 168 h (Q7) fermentation. **(B)** The main proteins secreted by *T. reesei* after 72 h of cultivation. **(C)** The main proteins secreted by *T. reesei* after 168 h of cultivation.

As mentioned above, there was a significant difference in the protease activities between 72 h (Q3) and 168 h (Q7). To determine the proteases in the Q3 and Q7 secretome, the total extracellular proteins from the culture supernatants were identified by LC-MS/MS. All proteins identified from Q3 and Q7 were listed in Supplementary Table S2 (Q3) and Supplementary Table S3 (Q7). The total number of extracellular proteins detected in Q3 and Q7 were 110 and 92, respectively. Among these proteins, 74 proteins were shared by Q3 and Q7, 36 proteins were unique in Q3, and 18 proteins were specific for Q7 (Figure 3A). It was known that the peptide spectrum match (PSM) values were often used to represent the protein contents (Wang et al., 2017). According to the PSMs, the 10 proteins with the highest contents in Q3 and Q7 were selected and shown in Figures 3B,C, respectively. The majority of these proteins were related to degradation of lignocellulose, including CBH1, CBH2, EG1, and EG2. Especially, the ratio of CBH1 (PSM, 798) to CBH2 (PSM, 227) was 3.5 in Q3, whereas that is 2.26 in Q7. These results indicated that the cellulase components were changed at different cultivation stages.

Production of proteases could be not only influenced by the composition of medium, in particular the organic nitrogen, but also regulated by the pH of culture media (Haab et al., 1990; Delgado-Jarana et al., 2000). In *T. reesei*, an aspartic acid protease was identified to be correlated with cellulase degradation at pH below 5.0 (Haab et al., 1990). When the pH was controlled at 6.0, the trypsin-like alkaline serine protease Tvp1 was considered as the major factor in degrading CBH1 (Dienes et al., 2007). In the present study, the pH of the fermentation supernatant in CSL condition was 4.0 and 6.5 at the early (Q3) and late (Q7) cultivation stages, respectively. Correspondingly, 12 proteases belonging to different families were identified (Table 1). Among these proteases, four proteases including the trypsin-like serine protease (tre73897), the aspartic protease (tre79807), the aminopeptidase (tre81070), and the zinc metallopeptidase (tre105808) were unique in Q3, whereas two serine proteases including the subtilase like protease (tre51365) and subtilisin-related protease (tre123234) were observed in Q7. In addition, six proteases (tre22459, tre77579, tre81517, tre103039, tre120998, and tre123244) belonging to aspartic protease, serine protease as well as carboxypeptidase were shared by Q3 and Q7. These results suggested that there was a significant difference in the protease components at different cultivation stages.

**TABLE 1:** The proteases in the fermentation supernatants of *T. reesei* identified by means of LC-MS/MS.

**Protein ID**	**Description**	**AAs**	**MW (KDa)**	**Calc. pI**	**PSMs (Q3)**	**PSMs (Q7)**
tre73897	Trypsin-like serine protease	259	24.6	5.85	4	
tre79807	Aspartyl protease	516	52.1	4.80	2	
tre81070	Aminopeptidase	513	53.4	4.69	2	
tre105808	Zinc metallopeptidases	314	32.2	5.04	1	
tre22459	Zinc carboxypeptidase	442	45.6	6.42	1	2
tre77579	Aspartyl protease	395	42.4	4.74	5	7
tre81517	Subtilisin	1419	151.9	7.37	1	5
tre103039	Serine-type peptidase	268	28.7	4.75	2	3
tre120998	Serine carboxypeptidase	548	59.2	5.08	1	2
tre123244	Serine protease-like protein	541	56.5	5.49	7	12
tre51365	Subtilase superfamily	882	91.4	5.08		1
tre123234	Subtilisin-related protease	391	38.9	5.83		1

*PSM indicates the peptide spectrum match.*

In the genome of *T. reesei*, approximately 200 genes involved in proteolytic degradation were annotated (Martinez et al., 2008). Furthermore, 39 proteases were found to be secreted and pH-dependent, which were determined by proteomics (Adav et al., 2011). Landowski et al. (2015) systematically identified 13 major proteases that are responsible for degradation of therapeutic proteins. More recently, Daranagama et al. (2019) found that the expression of trichodermapepsin (TrAsP) encoding-gene *trasp* is more favorable in acidic conditions in a galactose medium. These studies in combination with the above results demonstrated that *T. reesei* possesses a complex proteolytic system displaying different types of proteases in different growth conditions.

### Analysis of Protease Activity in the Individual Protease Gene-Deletion Strains

To determine which proteases were related to cellulase degradation, all the 12 protease-encoding genes identified by comparative secretomics were individually deleted in *T. reesei* QM9414Δ*mus53*. PCR analysis revealed that each protease gene was indeed deleted without ectopic integration of the deletion cassette (data not shown). When these protease gene-deletion strains were grown using the different protein-including nitrogen sources, including peptone, milk, gelatin and casein, the growth rates of most of the protease gene-deletion strains were similar to that of the parental strain QM9414Δ*mus53* (Supplementary Figure S1), except that deletion of *tre123244* resulted in poor growth and no sporulation (data not shown, and this strain was not investigated for further study). Similarly, these protease gene-deletion strains also did not display significant difference in the growth rate from that of the parental strain on the tested carbon sources, including glucose, lactose, and glycerol (Supplementary Figure S2). The phenomenon that the protease-gene-deletion stains could grow normally or even faster on the complex media has also been observed previously (Landowski et al., 2015). The possible explanations for the normal or improved growth could be related to the enhanced stability of extracellular cellulases that can faster utilize sugars and thus facilitate the fungal growth (Landowski et al., 2015). Taken together, these results illustrated that most of the protease-gene deletions did not affect fungal growth on nitrogen sources or carbon sources in *T. reesei*.

**FIGURE 4:**
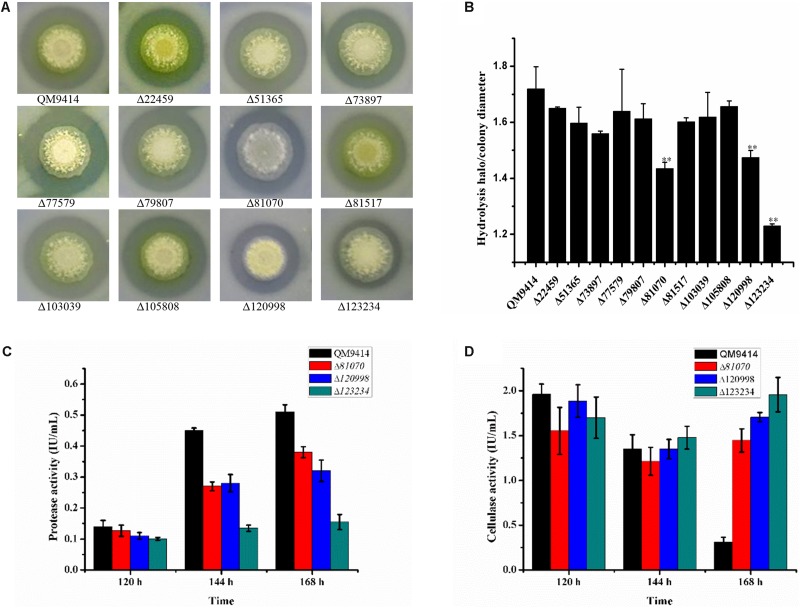
Evaluation of the ability to produce proteases by the protease gene-deletion strains in *T. reesei*. **(A)** The 11 protease gene-deletion strains were grown on the media containing 1% skim milk. The halo size around the fungal colony reflected the extent of the milk hydrolysis due to the protease production. **(B)** The ratios of hydrolysis halo to colony diameter presented by panel **(A)**. ^∗∗^*P* < 0.01. **(C)** Protease activities produced by the *T. reesei* protease gene-deletion strains Δ*tre81070*, Δ*tre120998*, Δ*tre123234* and the parental strain QM9414Δ*mus53*. **(D)** Protease activities produced by the *T. reesei* protease gene-deletion strains Δ*tre81070*, Δ*tre120998*, Δ*tre123234* and the parental strain QM9414Δ*mus53*.

To investigate the capacity to secrete proteases, these strains were cultured on the MM agar plates containing 1.0% skim-milk as the nitrogen substrate. It was found that these deletion strains exhibited the relatively smaller proteolytic halos around the colonies than that of the parental strain QM9414Δ*mus53* (Figure 4A). Among these strains, Δ*tre81070*, Δ*tre120998*, and Δ*tre123234* exhibited the smallest halos. In accordance with this result, the ratios of proteolytic halo to colony diameter in the three strains were also the lowest ones (Figure 4B). These results clearly revealed that deletion of *tre123234*, *tre120998* or *tre81070* significantly decreased the proteolytic capacities of *T. reesei*, demonstrating that tre81070, tre120998, and tre123234 are probably the major extracellular proteases related to the decreased activity of cellulase in *T. reesei.*

To further investigate the protease activity of Δ*tre81070*, Δ*tre120998*, and Δ*tre123234* during the fermentation for cellulase production, the deletion strains were cultured in the CPM)at 30°C for 7 days. The culture supernatants were collected for the extracellular protease detection. As expected, Δ*tre81070*, Δ*tre120998*, and Δ*tre123234* exhibited much lower protease activity than that of the parental strain QM9414Δ*mus53* (Figure 4C). On the same time, the three mutants also produced relatively higher cellulase activities at late cultivation stages among the protease gene-deletion mutants (Figure 4D). Especially, the protease activity of Δ*tre123234* showed a decrease of 55% in comparison with that of QM9414Δ*mus53* using CSL as the nitrogen source at late fermentation stages. These results further suggested that the three extracellular proteases, tre81070, tre120998, and tre123234, contribute to the major protease activity in *T. reesei* during the fermentation in CPM medium containing CSL as the nitrogen source.

Serine proteases have been reported as the key factor for extracellular protein degradation in *T. reesei* (Landowski et al., 2015). Deletion of the serine protease gene *spw1* (*tre121495*) resulted in a decrease on total extracellular protease activity (Zhang et al., 2014). Then the other two serine proteases SLP1 (tre51365) and TSP1 (tre73897) were later identified to be most critical for degradation of antibody heavy chain (Landowski et al., 2015). However, Spw1 was not detected in this study while deletion of Δ*tre51365* or Δ*tre73897* resulted in only a slight decrease in the protease activity, indicating the existence of additional serine proteases to support the extracellular protease activity. Here, tre123234 was found to be a serine protease at the late stage of fermentation and its gene deletion resulted in a 55% decrease in protease activity, demonstrating that tre123234 is probably the major serine protease related to cellulase degradation in *T. reesei*. In our previous study, the GATA transcription factor Are1 was shown to play an important role in the regulation of proteases (Qian et al., 2019). Especially, the protease tre123244 was found to be the target protease that was regulated by Are1, and there were the conserved Are1-binding sites in the promoter region of *tre123244*. Thus, it is speculated that tre123244 may be regulated by nitrogen sources. Furthermore, we inspected the yeast genome database with TBLASTX by using the sequence of tre123244 as the query. It was found that tre123244 has a high identity with the vacuole protease RB1 (NCBI YEL060C). PRB1 is shown to be involved in the proteolytic processing of the precursor proteins (Ogrydziak, 1993; Kerstens and Dijck, 2018). Therefore, it could be supposed that tre123244 is a vacuole protease and plays a key role in the basic cell metabolism.

### Simultaneous Disruption of the Triple Protease Genes in *T. reesei*

**FIGURE 5:**
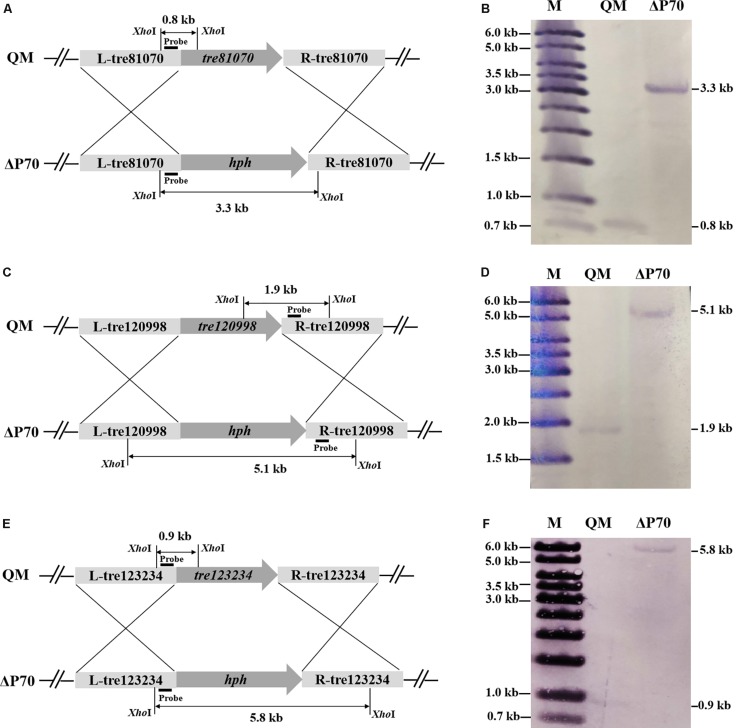
Southern blot analysis of the triple protease-gene deletions in *T. reesei* ΔP70. **(A)** Graphical representation of the genomic loci for the *tre81070* deletion in *T. reesei* ΔP70 and the parental strain QM9414Δ*mus53* (QM). **(B)** Southern blot of the *Xho*I-digested genomic DNA using a probe for detection of the *tre81070* deletion event. A fragment of 0.8 kb is present in the parental strain while a 3.3 kb band is shown in ΔP70. **(C)** Graphical representation of the genomic loci for the *tre120998* deletion in *T. reesei* ΔP70 and QM9414Δ*mus53* (QM). **(D)** Southern blot of the *Xho*I-digested genomic DNA using a probe for detection of the *tre120998* deletion event. A fragment of 1.9 kb is present in the parental strain while a 5.1 kb band is shown in ΔP70. **(E)** Graphical representation of the genomic loci for the *tre123234* deletion in *T. reesei* ΔP70 and QM9414Δ*mus53* (QM). **(F)** Southern blot of the *Xho*I-digested genomic DNA using a probe for detection of the *tre123234* deletion event. A fragment of 0.9 kb is present in the parental strain while a 5.8 kb band is shown in ΔP70.

**FIGURE 6:**
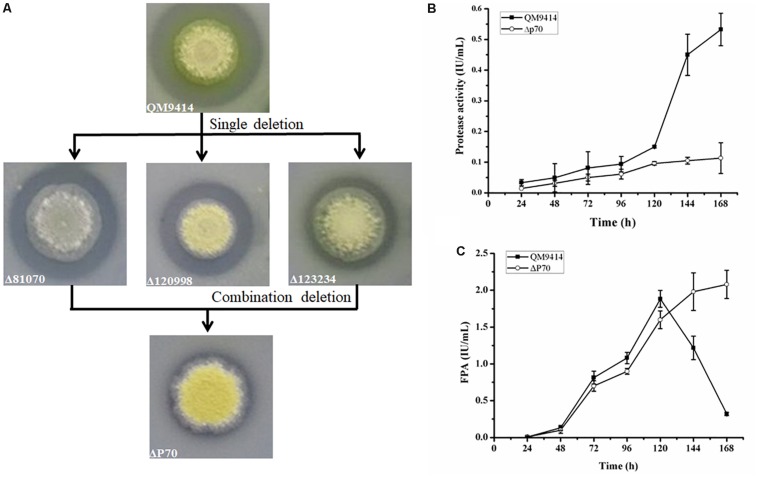
Production of protease and cellulase by the triple protease gene-deletion strain ΔP70. **(A)** The *T. reesei* protease gene-deletion strains were grown on the medium containing 1.0% skim milk. **(B)** The protease activities produced by *T. reesei* ΔP70. **(C)** The total cellulase activity (FPA) produced by *T. reesei* ΔP70. The values show the means of three biological replicates, and the error bars indicate the standard deviations.

Construction of industrially useful filamentous fungi require efficient genetic tools to perform time-saving sequential manipulations or multiplex manipulations. It was previously reported that the efficiency of homologous DNA integration in the *T. reesei mus53* deletion strain was up to 100% in some cases (Steiger et al., 2011; Chum et al., 2017). This inspired us to suppose that homologous DNA integration into multiple genomic loci may occur in QM9414Δ*mus53*. Thus, the selectable marker *hph* was used to simultaneously target three protease genes, *tre81070*, *tre120998*, and *tre123234*. After co-transformation of these three gene knock-out cassettes at the same molar concentration into the protoplasts of QM9414Δ*mus53*, 80 transformants were selected from the MM plates containing 300 μg/μL hygromycin B. Then these strains were verified through PCR analysis using the corresponding primer pairs (Supplementary Figures S3b–d). Among 80 hygromycin-resistant transformants, there were 32 single-gene-deletion transformants, 20 double-gene-deletion transformants for Δ*tre81070*/Δ*tre120998*, 10 double-gene-deletion transformants for Δ*tre81070*/Δ*tre123234*, 16 double-gene-deletion transformants for Δ*tre120998*/Δ*tre123234*, and two transformants with triple gene deletions (namely, ΔP70 and ΔP57). Although the frequency of triple deletions was quite low (2/80, i.e., 2.5%), QM9414Δ*mus53* is a promising host to target multiple genes simultaneously at the genome. The triple gene-deletion strain ΔP70 was further confirmed by Southern blot analysis. For the *tre81070* gene deletion, hybridization of *Xho*I-digested genomic DNA with the *tre81070* probe resulted in a 0.8 kb fragment in the parental strain QM9414Δ*mus53* while a 3.3 kb fragment in the ΔP70 strain (Figures 5A,B). For the *tre120998* gene deletion, hybridization with the *tre120998* probe gave a 1.9 kb fragment in QM9414Δ*mus53* and a 5.1 kb fragment in ΔP70 (Figures 5C,D). For the *tre123234* gene deletion, Southern hybridization produced a 0.9 kb fragment for QM9414Δ*mus53* and a 5.8 kb fragment for ΔP70 (Figures 5E,F). These results demonstrated that the three protease genes, *tre81070*, *tre120998*, and *tre123234*, were successfully knocked out in the three-deletion strain ΔP70. Subsequently, the ΔP70 strain was cultivated on the skim milk agar plate to test the ability to hydrolyze protein substrates (Figure 6A). The proteolytic halo around the colony of ΔP70 was much smaller than those of Δ*tre81070*, Δ*tre120998*,Δ*tre123234*, and QM9414Δ*mus53*. Furthermore, ΔP70 and the parental strain QM9414Δ*mus53* were fermented in CPM for 7 days, and the fermentation broths were used for enzyme activity assay. It was found that ΔP70 exhibited a 78% decrease in the protease activity at late fermentation stages (168 h) (Figure 6B). These results illustrated that simultaneous disruption of the three protease genes, *tre81070*, *tre120998*, and *tre123234*, resulted in dramatic decrease of extracellular protease activity in *T. reesei*.

### Cellulase Production by the Triple-Protease Gene-Deletion Strain ΔP70

**FIGURE 7:**
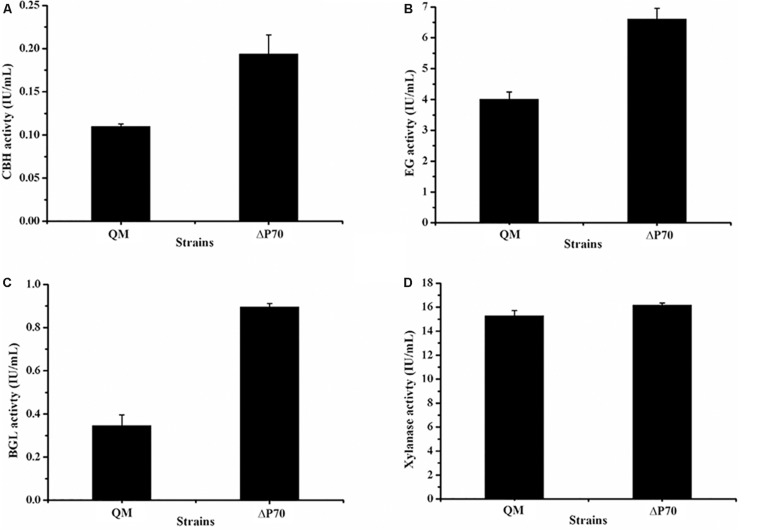
The activities of lignocellulolytic component enzymes produced by the triple protease gene-deletion strain ΔP70. **(A)** The endo-β-1,4-glucanase (EG) activity. **(B)** The cellobiohydrolase (CBH) activity. **(C)** The β-glucosidase (BGL) activity. **(D)** The xylanase activity. The values show the means of three biological replicates, and the error bars indicate the standard deviations.

Recently, Landowski et al. succeeded in decreasing the major extracellular proteases of *T. reesei* to make it as a more suitable system for therapeutic protein production (Landowski et al., 2015). However, after multiple protease deletions the total secreted protein was enhanced only around 22%. This may suggest that there are more proteases in the culture supernatant that would need to be deleted to improve native cellulase production. Therefore, the cellulase production by the triple deletion strain ΔP70 was performed in CPM for 168 h, and the fermentation broths were used for cellulase activity assay. As shown in Figure 6C, the FPA of ΔP70 reached 2.08 IU/mL, which was six-fold higher than that of QM9414Δ*mus53* (0.32 IU/mL), suggesting that degradation of cellulases was significantly alleviated at late fermentation stages in the triple-protease gene-deletion strain ΔP70. The low-protease-level strains obtained recently by the genetic modification methods in *T. reesei* were compared for the effect on cellulase/protein production (Supplementary Table S4). In addition, the β-glucosidase (BGL), CBH, EG, and xylanase activities in ΔP70 exhibited 150%, 75%, 65% and 6% higher than those of the parental strain QM9414Δ*mus53*, respectively (Figure 7). Taken together, these results demonstrated that disruption of the major extracellular protease genes significantly increased cellulase production in *T. reesei.*

## Conclusion

In this study, the cellulase activity produced by *T. reesei* was found to dramatically decreased at the late culture stages. Comparative secretomics strategy, combinated with multiplex gene manipulations, revealed the correlation between occurence of extracellular proteases and degradation of cellulases. The triple protease gene-deletion strain ΔP70 is sufficient to support the high-level cellulase production. These results indicate that deletion of multiple protease genes is a feasible strategy for improvement of cellulase production to enhance bioconversion efficiency of lignocellulosic biomass.

## Data Availability Statement

All datasets generated for this study are included in the article/Supplementary Material.

## Author Contributions

YuQ and YZ conceived the work, drafted the manuscript, performed the experiments, and analyzed the data. LiZ, NS, and YS participated in the experiments and collected the data. LeZ, WL, YiQ, and YZ designed the work and revised the manuscript. All authors read and approved the final version of the manuscript.

## Conflict of Interest

The authors declare that the research was conducted in the absence of any commercial or financial relationships that could be construed as a potential conflict of interest.

## Footnotes

1 http://genome.jgi-psf.org/Trire2

2 https://www.ebi.ac.uk/merops/
