# Supplementary material for: Enhancement of Cellulase Production in Trichoderma reesei via Disruption of Multiple Protease Genes Identified by Comparative Secretomics
Source: Front Microbiol. 2019 Dec 3;10:2784. doi: 10.3389/fmicb.2019.02784 (PMC6901835; doi:10.3389/fmicb.2019.02784)
Supplement: Supplementary file 1 [file Data_Sheet_1.docx]

**Additional file**

**Table S1** Oligonucleotides used in this study.

| **Primers** | **Sequences (5’-3’)** | **Employment** |
| --- | --- | --- |
| 22459-2117-UF1 | CACTATGCTCAGGGACTG | Construction and verification of Δ*tre22459* |
| 22459-498-UR1 | TGCTCCTTCAATATCAGTTAAGGTCGTATCACGGCTGGAGGTTGG | Construction and verification of Δ*tre22459* |
| 22459-217-DF1 | AAATTCCGTCACCAGCCCTGGGTTGTCGCTCGTTAGCAACCACT | Construction and verification of Δ*tre22459* |
| 22459-1744-DR1 | ACTATTACAAGTTCCTCGGT | Construction and verification of Δ*tre22459* |
| 22459-2093UF2 | CGAGAAATCGCAGGAGAAAA | Construction and verification of Δ*tre22459* |
| 22459-1657-DR2 | CAGACAGAAATCCAAACAATCC | Construction and verification of Δ*tre22459* |
| 51365-1258UF1 | CGATAGCGTCAGGAGACCA | Construction and verification of Δ*tre51365* |
| 51365-15-UR1 | TGCTCCTTCAATATCAGTTAAGGTCGCCGCTTGTCTGACTGTGGG | Construction and verification of Δ*tre51365* |
| 51365-11-DF1 | AAATTCCGTCACCAGCCCTGGGTTGGAGGGTTTCTTGAGGGGTAA | Construction and verification of Δ*tre51365* |
| 51365-1285-DR1 | ATGCGCAGCTTGCGATAGAG | Construction and verification of Δ*tre51365* |
| 51365-1176-UF2 | CTCGGAGTGATGCTTCCTTTG | Construction and verification of Δ*tre51365* |
| 51365-1239-DR2 | GCTGGGCAGGCTCTTTGAC | Construction and verification of Δ*tre51365* |
| 73897-1510-UF1 | ATCTTGTCGTTATCGCTCCC | Construction and verification of Δ*tre73897* |
| 73897-179-UR1 | CTCCTTCAATATCAGTTAAGGTCGCACCACAACAAGGCAACAAT | Construction and verification of Δ*tre73897* |
| 73897-464-DF1 | AAATTCCGTCACCAGCCCTGGGTTG TCTCAATAGGCTCCAACCC | Construction and verification of Δ*tre73897* |
| 73897-1854-DR1 | GATTGATAGCCGCTGGATT | Construction and verification of Δ*tre73897* |
| 73897-1083-UF2 | ACGGTTCGTGATAGGTTC | Construction and verification of Δ*tre73897* |
| 73897-1671-DR2 | TCCTGACTAATCTGACCGT | Construction and verification of Δ*tre73897* |
| 77579-1988-UF1 | ATCGTATCCTGTTACTGT | Construction and verification of Δ*tre77579* |
| 77579-295UR1 | TGCTCCTTCAATATCAGTTAAGGTCGCAGGTCGCATTTGGGGTG | Construction and verification of Δ*tre77579* |
| 77579-58-DF1 | AAATTCCGTCACCAGCCCTGGGTTGGCAGAATGCTGGGTAGTTT | Construction and verification of Δ*tre77579* |
| 77579-1935-DR1 | CTTCAGACGCCAGCTCTG | Construction and verification of Δ*tre77579* |
| 77579-1951-UF2 | CCCGATGGAGTGATGGACAG | Construction and verification of Δ*tre77579* |
| 77579-1869-DR2 | GGGAGGGTTAAGTTGTGGT | Construction and verification of Δ*tre77579* |
| 79807-1475-UF1 | GACCAGGCTTGAATATCA | Construction and verification of Δ*tre79807* |
| 79807-233-UR1 | TGCTCCTTCAATATCAGTTAAGGTCGACGAGATGCGGAGAATGA | Construction and verification of Δ*tre79807* |
| 79807-92-DF1 | AAATTCCGTCACCAGCCCTGGGTTGAGACGGACCCATTCCATTAC | Construction and verification of Δ*tre79807* |
| 79807-1807-DR1 | TCGCCAGCCACAGGTAGG | Construction and verification of Δ*tre79807* |
| 79807-1396-UF2 | TCAGGCAAAGTCGGGTTA | Construction and verification of Δ*tre79807* |
| 79807-1731-DR2 | CTTCAGACGCCAGCTCTG | Construction and verification of Δ*tre79807* |
| 81070-2081-UF1 | ATCTGGCTGACAGTATGGG | Construction and verification of Δ*tre81070* and the triple gene-deletion strains |
| 81070-221-UR1 | CTCCTTCAATATCAGTTAAGGTCGGTCACCGCTGCCTATCTTA | Construction and verification of Δ*tre81070* and the triple gene-deletion strains |
| 81070-193-DF1 | AAATTCCGTCACCAGCCCTGGGTTGTTCGGCTTCTTCTAACACTCACAT | Construction and verification of Δ*tre81070* and the triple gene-deletion strains |
| 81070-1916-DR1 | GGGCGACATGGATCAGCA | Construction and verification of Δ*tre81070* and the triple gene-deletion strains |
| 81070-1536-UF2 | ATGGCGATGAAAACCCAC | Construction and verification of Δ*tre81070* and the triple gene-deletion strains |
| 81070-1589-DR2 | AGCAGGATGCCCAGAACA | Construction and verification of Δ*tre81070* and the triple gene-deletion strains |
| 81070-551-F1 | CCTGGTGCTCGTCCAAAAC | Construction and verification of Δ*tre81070* and the triple gene-deletion strains |
| 81070-1699-R1 | GCAACCGAGTGGGCAATAA | Construction and verification of Δ*tre81070* and the triple gene-deletion strains |
| 81517-1632-UF1 | CCACTTACTGTACATCCATG | Construction and verification of Δ*tre81517* |
| 81517-21-UR1 | TGCTCCTTCAATATCAGTTAAGGTCGGTGCTGAGGGAGCAGGAC | Construction and verification of Δ*tre81517* |
| 81517-65-DF1 | AAATTCCGTCACCAGCCCTGGGTTGCCTGGCATCAATTTCGGTC | Construction and verification of Δ*tre81517* |
| 81517-1514-DR1 | TGCCTGCTGTGTCGTCTG | Construction and verification of Δ*tre81517* |
| 81517-1597-UF2 | TCCGTCATTTCTGCCCATC | Construction and verification of Δ*tre81517* |
| 81517-1476-DR2 | TGCGGCAGCAGTCAGTAT | Construction and verification of Δ*tre81517* |
| 103039-1692-UF1 | CGGCACTTATGATGTTGGTA | Construction and verification of Δ*tre103039* |
| 103039-2-UR1 | TGCTCCTTCAATATCAGTTAAGGTCGGTATTGAATGGCGGTCGG | Construction and verification of Δ*tre103039* |
| 103039-283-DF1 | AAATTCCGTCACCAGCCCTGGGTTGCGAAGATGCTGCCTCCCA | Construction and verification of Δ*tre103039* |
| 103039-1620-DR1 | GATACTGTCGCTCCCTTCG | Construction and verification of Δ*tre103039* |
| 103039-1641-UF2 | GCTGCTTGAAGTAGAACGAGG | Construction and verification of Δ*tre103039* |
| 103039-1528-DR2 | GCAACCTCCGAATCGTGAC | Construction and verification of Δ*tre103039* |
| 105808-1790-UF1 | CAAGCTCATAGTTACATA | Construction and verification of Δ*tre105808* |
| 105808-132-UR1 | TGCTCCTTCAATATCAGTTAAGGTCGGCCGTAGAGGTCCTGAAGC | Construction and verification of Δ*tre105808* |
| 105808-188-DF1 | AAATTCCGTCACCAGCCCTGGGTTGTGGGGTCACGAGTGTTGTAT | Construction and verification of Δ*tre105808* |
| 105808-1805-DR1 | GACGAACACAGGGGCTTCG | Construction and verification of Δ*tre105808* |
| 105808-1763-UF2 | CAAACATACCCTGTCTCCCC | Construction and verification of Δ*tre105808* |
| 105808-1723-DR2 | GAGGGCTTGATGTCTTATGG | Construction and verification of Δ*tre105808* |
| 120998-2086-UF1 | AAGACCCCTACTTCCGTT | Construction and verification of Δ*tre120998* and the triple gene-deletion strains |
| 120998-381-UR1 | CTCCTTCAATATCAGTTAAGGTCGGTGTTTGTCACTGCTGGAT | Construction and verification of Δ*tre120998* and the triple gene-deletion strains |
| 120998-151-DF | AAATTCCGTCACCAGCCCTGGGTTGGATTCGTTGATGGATGACTA | Construction and verification of Δ*tre120998* and the triple gene-deletion strains |
| 120998-2005-DR1 | TAACAGAAAGCACCCGAC | Construction and verification of Δ*tre120998* and the triple gene-deletion strains |
| 120998-1545-UF2 | GGACGAGGTTGTGAAGCA | Construction and verification of Δ*tre120998* and the triple gene-deletion strains |
| 120998-1277-DR2 | GCCCTGGTACACTGACACTC | Construction and verification of Δ*tre120998* and the triple gene-deletion strains |
| 120998-294-F1 | AAGCAGTTTAGCGGATACCT | Construction and verification of Δ*tre120998* and the triple gene-deletion strains |
| 120998-1437-R1 | TGTCGCCAACGGTAAGAT | Construction and verification of Δ*tre120998* and the triple gene-deletion strains |
| 123234-1995-UF1 | GCTCACCTACCGTAACTCA | Construction and verification of Δ*tre123234* and the triple gene-deletion strains |
| 123234-197-UR1 | CTCCTTCAATATCAGTTAAGGTCGAATAACCACCTCTTCATCG | Construction and verification of Δ*tre123234* and the triple gene-deletion strains |
| 123234-421-DF1 | AAATTCCGTCACCAGCCCTGGGTTGTCCCGACTCAGGCAATCCA | Construction and verification of Δ*tre123234* and the triple gene-deletion strains |
| 123234-2003-DR1 | GACCAAAAGGCGGAACACG | Construction and verification of Δ*tre123234* and the triple gene-deletion strains |
| 123234-1334-UF2 | CAACTATGCGAGTGAATGGG | Construction and verification of Δ*tre123234* and the triple gene-deletion strains |
| 123234-1615-DR2 | CCAAAGCGGCACTCATTAC | Construction and verification of Δ*tre123234* and the triple gene-deletion strains |
| 123234-215-F1 | ATGTCTTCCGTGGGTTTGC | Construction and verification of Δ*tre123234* and the triple gene-deletion strains |
| 123234-1206-R1 | GCCGACGATGTGAGGAGTG | Construction and verification of Δ*tre123234* and the triple gene-deletion strains |
| 123244-1820-UF1 | GCTCGTAGCCAAGTAGGG | Construction and verification of Δ*tre123244* |
| 123244-78-UR1 | CTCCTTCAATATCAGTTAAGGTCGAAAACGCAGCGAAACAGA | Construction and verification of Δ*tre123244* |
| 123244-325-DF1 | AAATTCCGTCACCAGCCCTGGGTTGCCCAATCCTCGCAATCTT | Construction and verification of Δ*tre123244* |
| 123244-1557-DR1 | GGTCGCCATTATCCGTTG | Construction and verification of Δ*tre123244* |
| 123244-1458-UF2 | ACGGCAGGACAGGACGAATA | Construction and verification of Δ*tre123244* |
| 123244-1287-DR2 | TCTCAAACGACGGGAGGGAC | Construction and verification of Δ*tre123244* |
| hph-F1 | CGACCTTAACTGATATTGAAGGAG | Amplification of the *hph* gene |
| hph-R1 | CAACCCAGGGCTGGTGAC | Amplification of the *hph* gene |
| Y-hph-121-DF1 | ACTGAGGAATCCGCTCTTGG | Verification of the gene deletion |
| Y-hph-168-UR1 | ACTGCTTACAAGTGGGCTGA | Verification of the gene deletion |
| Probe-81070-F | CGACGCTGAGAAGAAAGACT | Preparation of the probe for *tre81070* |
| Probe-81070-R | GTCACCGCTGCCTATCTTAC | Preparation of the probe for *tre81070* |
| Probe-120998-F | CGAGGTCAGGAGGACATAAA | Preparation of the probe for *tre120998* |
| Probe-120998-R | CAAGCAGGAGATTCAAGTCAT | Preparation of the probe for *tre120998* |
| Probe-123234-F | GTGACTATCCTGGGGTGGTA | Preparation of the probe for *tre123234* |
| Probe-123234-R | GACTATGGTGGTTTGGGAAG | Preparation of the probe for *tre123234* |

**Table S2** The secreted proteins identified by means of LC-MS/MS from the fermentation supernatants of *T. reesei* grown in CSL medium after 72 h of cultivation.

| **Protein ID** | **PSMs** | **AAs** | **MW [kDa]** | **Calc. pI** | **Description** | |
| --- | --- | --- | --- | --- | --- | --- |
| 123989 | 798 | 433 | 45.8 | 4.5 | CBH1, GH7 | |
| 121136 | 302 | 413 | 44.3 | 6.52 | Hypothetical protein-transmembrane | |
| 73631 | 259 | 534 | 56.9 | 6.1 | Isoamyl alcohol oxidase | |
| 72567 | 227 | 471 | 49.6 | 5.34 | CBH2, GH6 | |
| 123818 | 177 | 223 | 24.1 | 8.27 | Xylanase 2, GH11 | |
| 122081 | 162 | 453 | 47.4 | 4.94 | EG1, GH7 | |
| 22386 | 88 | 232 | 24.2 | 4.68 | Hypothetical protein | |
| 49081 | 63 | 838 | 87.1 | 5.78 | Xyloglucanase, GH74 | |
| 120312 | 60 | 418 | 44.1 | 5.22 | EG2, GH5 | |
| 123967 | 58 | 102 | 10.4 | 6.33 | Fungal hydrophobin HFB3 | |
| 104664 | 57 | 419 | 43.5 | 5.16 | Putative cell wall protein | |
| 70840 | 56 | 195 | 18.7 | 7.5 | Hypothetical protein | |
| 123992 | 54 | 493 | 51.5 | 5.02 | Swollenin, N-terminal CBM1 module | |
| 74060 | 51 | 283 | 28.1 | 5.17 | Hypothetical protein | |
| 104401 | 37 | 195 | 18.7 | 4.41 | Putative cell wall protein with a CFEM domain | |
| 1885 | 31 | 631 | 67.2 | 5.3 | Glucan-1,4-α-glucosidase with starch-binding domain | |
| 123283 | 31 | 499 | 51 | 6.52 | α-L-arabinofuranosidase B, GH54 | |
| 55319 | 30 | 510 | 53.1 | 6.11 | Candidate α-L-arabinofuranosidase, GH54 | |
| 123232 | 29 | 198 | 21.4 | 7.27 | EG3, GH12 | |
| 119085 | 26 | 219 | 21.3 | 4.94 | Hypothetical protein | |
| 23115 | 25 | 257 | 26.4 | 5.03 | Distantly related to plant expansins, EXPN | |
| 124282 | 24 | 1090 | 114 | 4.83 | Cro1 | |
| 22914 | 23 | 473 | 50.8 | 4.81 | β-1,3-glucanosyltransglycosylase | |
| 74282 | 23 | 705 | 78.9 | 8.09 | Proteins containing Ca2+-binding EGF-like domains | |
| 73638 | 19 | 234 | 24.8 | 4.68 | Cip1 | |
| 122127 | 19 | 172 | 18.4 | 8.32 | Hypothetical protein | |
| 65406 | 19 | 269 | 28.4 | 4.96 | GH16 | |
| 68064 | 18 | 503 | 55.1 | 4.58 | GH43 | |
| 121127 | 15 | 788 | 86.2 | 5.87 | β-xylosidase, GH3 | |
| 119568 | 15 | 241 | 24.3 | 5.92 | Ca2+-modulated nonselective cation channel polycystin | |
| 111915 | 14 | 143 | 15.3 | 6.25 | Hypothetical protein | |
| 76155 | 13 | 396 | 44.4 | 5.76 | Acid phosphatase-like protein | |
| 121746 | 13 | 776 | 83 | 6.25 | Candidate exo-1,3-b-glucanase, GH55 | |
| 112286 | 10 | 254 | 26 | 7.02 | Hypothetical protein | |
| 69276 | 10 | 438 | 46.4 | 7.4 | β-glucocerebrosidase, GH30 | |
| 54242 | 10 | 716 | 74.8 | 5.5 | Candidate β-1,3-glucanase, GH55 | |
| 123726 | 10 | 268 | 29 | 5.27 | Candidateβ-1,3-1,4-glucanase, GH16 | |
| 73643 | 10 | 258 | 26.9 | 4.87 | Cel61A, AA9, GH61 |  |
| 72379 | 10 | 893 | 96.9 | 4.75 | Hypothetical protein | |
| 66792 | 9 | 319 | 34.5 | 5.1 | Candidate β-glycosidase, GH17 | |
| 120961 | 8 | 239 | 25.8 | 7.42 | Cel61B, AA9, GH61 | |
| 73523 | 8 | 803 | 84.6 | 4.56 | Hypothetical peroxidases | |
| 123244 | 7 | 515 | 55 | 6.2 | Serine protease-like protein | |
| 123084 | 7 | 262 | 27.9 | 7.53 | Dehyrdogenase/reductase domain-containing protein | |
| 79448 | 6 | 173 | 18.2 | 5.19 | Carbamoyl-phosphate synthase L chain, ATP-binding | |
| 123039 | 6 | 237 | 22.4 | 4.79 | Cell wall protein with a CFEM domain | |
| 123659 | 6 | 188 | 19.6 | 5.03 | Putative cell wall protein. | |
| 69245 | 6 | 957 | 106.7 | 4.81 | Candidate β-mannosidase, GH2 | |
| 77579 | 5 | 395 | 42.4 | 4.89 | Aspartyl protease | |
| 73632 | 5 | 206 | 20.9 | 5.31 | Axe1, CE5 | |
| 56996 | 5 | 344 | 37.7 | 5.11 | Man5a, mannanendo-1,4-β-mannosidase | |
| 71094 | 4 | 352 | 38.2 | 4.73 | Intradiol ring-cleavage dioxygenase | |
| 11963 | 4 | 181 | 18.1 | 4.77 | Anchored protein | |
| 124277 | 4 | 238 | 24.4 | 4.92 | Hypothetical protein | |
| 82633 | 4 | 397 | 43.1 | 4.81 | GH2 | |
| 81778 | 4 | 679 | 75.4 | 5.29 | Hypothetical protein | |
| 72632 | 4 | 404 | 44 | 5.03 | α-galactosidase 1, GH27 | |
| 76971 | 4 | 235 | 24.7 | 4.97 | H+-transporting two-sector ATPase | |
| 44214 | 4 | 245 | 25.1 | 4.59 | Axe2, candidate acetyl xylanesterase, CE5 | |
| 120821 | 4 | 274 | 27.8 | 5.78 | Hypothetical protein | |
| 21960 | 4 | 618 | 68.2 | 5.17 | Hospholipase C | |
| 73897 | 4 | 252 | 25.8 | 6.28 | Trypsin-like serine protease | |
| 55887 | 3 | 356 | 38.1 | 6.34 | Hypothetical protein CIMG | |
| 53947 | 3 | 165 | 16.2 | 5.3 | SWI-SNF chromatin-remodeling complex protein | |
| 82235 | 3 | 858 | 95.4 | 6.39 | Candidate α-glucosidase, GH31 | |
| 122487 | 3 | 158 | 16.9 | 4.7 | Hypothetical protein | |
| 76672 | 3 | 744 | 78.4 | 6.86 | Cel3A, BGL1, GH3 | |
| 109907 | 3 | 206 | 21.5 | 7.44 | Citrate binding protein | |
| 22915 | 3 | 469 | 50.7 | 5.21 | Glucose oxidase | |
| 80833 | 3 | 424 | 46 | 5.68 | Chitinase, GH18 | |
| 123955 | 2 | 142 | 15 | 4.79 | Hypothetical protein bearing a cerato-platanin domain | |
| 55443 | 2 | 119 | 12.6 | 5.45 | Predicted protein | |
| 81070 | 2 | 371 | 40 | 4.81 | Aminopeptidase | |
| 104461 | 2 | 343 | 37.1 | 5.57 | Soluble epoxide hydrolase | |
| 111849 | 2 | 473 | 52.8 | 6.07 | Xylanase 4, GH5 | |
| 103039 | 2 | 286 | 30.6 | 5.1 | Peptidase S51, dipeptidase E | |
| 21468 | 2 | 272 | 27 | 5.05 | Fasciclin and related adhesion glycoprotein | |
| 82662 | 2 | 119 | 12.3 | 6.24 | Hypothetical protein | |
| 65483 | 2 | 190 | 20 | 8.68 | Hypothetical protein | |
| 104079 | 2 | 151 | 16 | 6.14 | Distantly related to plant expansins, EXPN | |
| 79807 | 2 | 387 | 40.4 | 5.11 | Aspartyl protease | |
| 120908 | 2 | 156 | 17.2 | 5.35 | Hypothetical protein | |
| 122374 | 2 | 120 | 13.2 | 8.6 | Predicted protein | |
| 123538 | 2 | 410 | 44.1 | 5 | β-1,3-glucanosyltransglycosylase, GH72 | |
| 105808 | 1 | 314 | 33.9 | 5.41 | Peptidase M, neutral zinc metallopeptidases | |
| 38441 | 1 | 347 | 35.2 | 4.41 | Hypothetical protein CIMG | |
| 121739 | 1 | 116 | 12.4 | 4.54 | Predicted protein | |
| 124198 | 1 | 127 | 14 | 8.02 | Hypothetical protein, CIMG | |
| 42152 | 1 | 227 | 24 | 4.65 | Candidate chitosanase, GH75 | |
| 108672 | 1 | 583 | 61.1 | 5.05 | Candidate α-1,3-glucanase, GH71 | |
| 120229 | 1 | 320 | 35.2 | 8.37 | Xylanase III, GH10 | |
| 22459 | 1 | 393 | 42.2 | 7.09 | Peptidase M14, carboxypeptidase A | |
| 63526 | 1 | 157 | 16.9 | 4.7 | Hypothetical protein | |
| 57098 | 1 | 779 | 88 | 5.24 | Hypothetical protein | |
| 124256 | 1 | 570 | 62 | 4.97 | Phospholipase-like protein | |
| 105237 | 1 | 182 | 20 | 5.25 | Hypothetical protein | |
| 70845 | 1 | 763 | 79.3 | 6.35 | Candidate β-1,3-glucanase, GH55 | |
| 105275 | 1 | 504 | 51.2 | 5.57 | Hypothetical protein | |
| 110894 | 1 | 478 | 52.2 | 6.24 | Candidate endo-β-1,6-galactanase, GH5 | |
| 39588 | 1 | 331 | 35.6 | 8.62 | Monodehydroascorbate/ferredoxin reductase | |
| 81517 | 1 | 515 | 55.5 | 5.82 | Peptidase S8 and S53 | |
| 49274 | 1 | 304 | 33.2 | 4.77 | GH16 | |
| 40830 | 1 | 301 | 32.3 | 6.73 | Hypothetical protein | |
| 71394 | 1 | 488 | 51.9 | 7.09 | β-glycosidases, GH79 | |
| 105874 | 1 | 697 | 78.5 | 6.28 | Proteins containing the FAD binding domain | |
| 66814 | 1 | 602 | 67.8 | 6.04 | Glycosyltransferase | |
| 120998 | 1 | 536 | 60.4 | 9.13 | Peptidase_S10, Serine carboxypeptidase | |
| 54632 | 1 | 487 | 52.9 | 6.32 | Major facilitator superfamily | |
| 111713 | 1 | 388 | 42.1 | 7.03 | Hypothetical protein | |
| 119989 | 1 | 71 | 7.2 | 6.92 | Hfb2, hydrophobin | |

**Table S3** The secreted proteins identified by LC-MS/MS from the fermentation supernatants of *T. reesei* grown in CSL medium after 168 h of cultivation.

| **Protein ID** | **PSMs** | **AAs** | **MW [kDa]** | **Calc. pI** | **Description** |
| --- | --- | --- | --- | --- | --- |
| 123989 | 744 | 433 | 45.8 | 4.5 | CBH1, GH7 |
| 72567 | 329 | 471 | 49.6 | 5.34 | CBH2, GH6 |
| 121316 | 195 | 413 | 44.3 | 6.52 | Hypothetical protein-transmembrane |
| 122081 | 152 | 459 | 48.2 | 4.94 | EG1, GH7 |
| 73631 | 88 | 534 | 56.9 | 6.1 | Isoamyl alcohol oxidase |
| 120312 | 83 | 418 | 44.1 | 5.22 | EG2, GH5 |
| 123232 | 65 | 198 | 21.4 | 7.27 | EG3, GH12 |
| 49081 | 60 | 838 | 87.1 | 5.78 | Xyloglucanase, GH74 |
| 123818 | 58 | 223 | 24.1 | 8.27 | Xylanase 2, GH11 |
| 123992 | 50 | 493 | 51.5 | 5.02 | Swollenin, N-terminal CBM1 module |
| 123283 | 44 | 499 | 51 | 6.52 | α-L-arabinofuranosidase B, GH54 |
| 22386 | 37 | 232 | 24.2 | 4.68 | Hypothetical protein |
| 70840 | 33 | 195 | 18.7 | 7.5 | Hypothetical protein |
| 55319 | 30 | 510 | 53.1 | 6.11 | Candidate α-L-arabinofuranosidase, GH54 |
| 120961 | 29 | 239 | 25.8 | 7.42 | Cel61b, AA9 |
| 76155 | 22 | 396 | 44.4 | 5.76 | Acid phosphatase-like protein |
| 120229 | 18 | 320 | 35.2 | 8.37 | Xylanase 3, GH10 |
| 104664 | 18 | 419 | 43.5 | 5.16 | Putative cell wall protein |
| 122127 | 18 | 172 | 18.4 | 8.32 | Hypothetical protein |
| 73638 | 16 | 234 | 24.8 | 4.68 | Cip1 |
| 65406 | 15 | 269 | 28.4 | 4.96 | GH16 |
| 121418 | 15 | 348 | 39.1 | 6.52 | Lipolytic enzyme |
| 123967 | 13 | 102 | 10.4 | 6.33 | Hfb3, Class II fungal hydrophobin, hfb3 |
| 111915 | 13 | 143 | 15.3 | 6.25 | Hypothetical protein |
| 124282 | 12 | 1090 | 114 | 4.83 | Cro1 |
| 123244 | 12 | 515 | 55 | 6.2 | Serine protease-like protein |
| 22914 | 10 | 473 | 50.8 | 4.81 | Candidate β-1,3-glucanosyltransglycosylase |
| 76672 | 9 | 744 | 78.4 | 6.86 | Cel3A, BGL1 |
| 1885 | 8 | 631 | 67.2 | 5.3 | Glucan-1,4-α-glucosidase with starch-binding domain |
| 73643 | 8 | 258 | 26.9 | 4.87 | Cel61a, AA9 |
| 123940 | 8 | 373 | 39.2 | 7.11 | Cip2 |
| 79448 | 7 | 173 | 18.2 | 5.19 | Carbamoyl-phosphate synthase L chain, ATP-binding |
| 56996 | 7 | 344 | 37.7 | 5.11 | Man5a, mannan endo-1,4-β-mannosidase |
| 74282 | 7 | 705 | 78.9 | 8.09 | Proteins containing Ca2+-binding EGF-like domains |
| 66792 | 7 | 319 | 34.5 | 5.1 | Candidate β-glycosidase, GH17 |
| 76210 | 7 | 312 | 33.7 | 6.92 | Abf2, Candidate a-L-arabinofuranosidase, GH62 |
| 77579 | 7 | 395 | 42.4 | 4.89 | Aspartyl protease |
| 119568 | 6 | 241 | 24.3 | 5.92 | Ca2+-modulated nonselective cation channel polycystin |
| 121127 | 6 | 788 | 86.2 | 5.87 | β-xylosidase, GH3 |
| 72632 | 6 | 404 | 44 | 5.03 | α-galactosidase 1, GH27 |
| 104661 | 5 | 343 | 37.1 | 5.57 | Soluble epoxide hydrolase |
| 119085 | 5 | 219 | 21.3 | 4.94 | Hypothetical protein |
| 111849 | 5 | 473 | 52.8 | 6.07 | Xylanase 4, GH5 |
| 119989 | 5 | 71 | 7.2 | 6.92 | Hfb2, hydrophobin |
| 81517 | 5 | 515 | 55.5 | 5.82 | Peptidase S8 and S53 |
| 69276 | 4 | 438 | 46.4 | 7.4 | β-glucocerebrosidase, GH30 |
| 73632 | 4 | 206 | 20.9 | 5.31 | axe1, acetyl xylan esterase, CE5 |
| 123039 | 4 | 237 | 22.4 | 4.79 | Putative cell wall protein with a CFEM domain |
| 120821 | 4 | 274 | 27.8 | 5.78 | Hypothetical protein |
| 21960 | 4 | 618 | 68.2 | 5.17 | Hospholipase C |
| 49976 | 4 | 229 | 23.3 | 4.88 | Endoglucanase V, GH45 |
| 122374 | 4 | 120 | 13.2 | 8.6 | Predicted protein |
| 76971 | 4 | 235 | 24.7 | 4.97 | H+-transporting two-sector ATPase |
| 55443 | 3 | 176 | 18.5 | 4.68 | Predicted protein |
| 104401 | 3 | 195 | 18.7 | 4.41 | Putative cell wall protein with a CFEM domain |
| 123659 | 3 | 89 | 9.7 | 4.73 | Putative cell wall protein |
| 108663 | 3 | 125 | 13.6 | 7.94 | Hypothetical protein |
| 68064 | 3 | 503 | 55.1 | 4.58 | GH43 |
| 65483 | 3 | 190 | 20 | 8.68 | Hypothetical protein |
| 44214 | 3 | 245 | 25.1 | 4.59 | Axe2 candidate acetyl xylan esterase, CE5 |
| 103039 | 3 | 286 | 30.6 | 5.1 | Peptidase S51, dipeptidase E |
| 102908 | 3 | 193 | 20.9 | 5.35 | Hypothetical protein |
| 21468 | 2 | 272 | 27 | 5.05 | Fasciclin and related adhesion glycoprotein |
| 4213 | 2 | 224 | 24.8 | 4.36 | Ribonuclease, T2 family |
| 123726 | 2 | 268 | 29 | 5.27 | Candidate β-1,3-1,4-glucanase, GH16 |
| 12286 | 2 | 251 | 25.7 | 6.67 | Hypothetical protein |
| 81778 | 2 | 679 | 75.4 | 5.29 | Hypothetical protein |
| 63526 | 2 | 157 | 16.9 | 4.7 | Hypothetical protein |
| 122870 | 2 | 218 | 21.3 | 5.1 | Putative cell wall protein |
| 112018 | 2 | 197 | 22.3 | 5.19 | Hypothetical protein |
| 22459 | 2 | 393 | 42.2 | 7.09 | Peptidase M14, carboxypeptidase A |
| 120998 | 2 | 548 | 60.9 | 5.31 | Peptidase_S10, Serine carboxypeptidase |
| 124277 | 1 | 238 | 24.4 | 4.92 | Hypothetical protein |
| 121746 | 1 | 752 | 80.6 | 6.25 | Candidate exo-1,3-β-glucanase, GH55 |
| 123234 | 1 | 387 | 40.1 | 6.44 | Proteinase T-like protein, Peptidase S8 and S53 |
| 54242 | 1 | 716 | 74.8 | 5.5 | Candidate β-1,3-glucanase, GH5 |
| 121739 | 1 | 116 | 12.4 | 4.54 | Predicted protein |
| 71094 | 1 | 352 | 38.2 | 4.73 | Intradiol ring-cleavage dioxygenase |
| 122487 | 1 | 158 | 16.9 | 4.7 | Hypothetical protein |
| 53947 | 1 | 165 | 16.2 | 5.3 | SWI-SNF chromatin-remodeling complex protein |
| 11963 | 1 | 181 | 18.1 | 4.77 | Anchored protein |
| 74060 | 1 | 283 | 28.1 | 5.17 | Hypothetical protein |
| 51365 | 1 | 824 | 87.1 | 5.29 | Subtilisin kexin isozyme-1/site 1 protease |
| 123084 | 1 | 255 | 27.1 | 6.87 | Dehyrdogenase/reductase domain-containing protein |
| 121475 | 1 | 687 | 75.8 | 5.08 | Hypothetical protein, CIMG |
| 76266 | 1 | 416 | 43.3 | 4.89 | GH16 |
| 72379 | 1 | 893 | 96.9 | 4.75 | Hypothetical proteinase |
| 74118 | 1 | 240 | 24.2 | 5.63 | TPR repeat-containing protein |
| 55887 | 1 | 356 | 38.1 | 6.34 | Hypothetical protein, CIMG |
| 112496 | 1 | 676 | 75.1 | 6.93 | Cytochrome cheme-binding site |
| 38441 | 1 | 347 | 35.2 | 4.41 | Hypothetical protein CIMG |
| 23115 | 1 | 257 | 26.4 | 5.03 | Distantly related to plant expansins, EXPN |

Table S4. The low-protease-level strains in *T. reesei*

| Strains | Engineering strategy | Protease activity | Effect on production | Reference |
| --- | --- | --- | --- | --- |
| RUT-C30 | Deletion of the alkaline serine protease SPW | Lost 55% of protease activity  at pH 7.0 and 44% activity at pH 6.0 | Increase 8 % of heterologous endoglucanase activity | Zhang et al., 2014 |
| QM6a | Deletion of seven-protease genes | Lost 96% of extracellular protease activity | Stable expression of heterologous protein, such as, heavy chain of antibody, interferon alpha 2b, and insulin like | Landowski et al., 2015 |
| QM6a | Deletions of nine-protease  genes | No reported | Increase heterologous IFNα-2b yields to 2.4 g/L, | Landowski et al., 2016 |
| QM9414 | Deletion of trichodermapepsin (TrAsP) | Lost 13% of protease activity | Neary increase 30% in Avicelase activity | Daranagama et al., 2019 |
| QM9414 | Deletion of three-protease genes | Lost 78% of protease activity | Increase 6-fold [homologous](D:/%E8%BD%AF%E4%BB%B6/office2007pro/%E6%9C%89%E9%81%93/Dict/7.5.2.0/resultui/dict/javascript:;) cellulase activity | In this study |

**
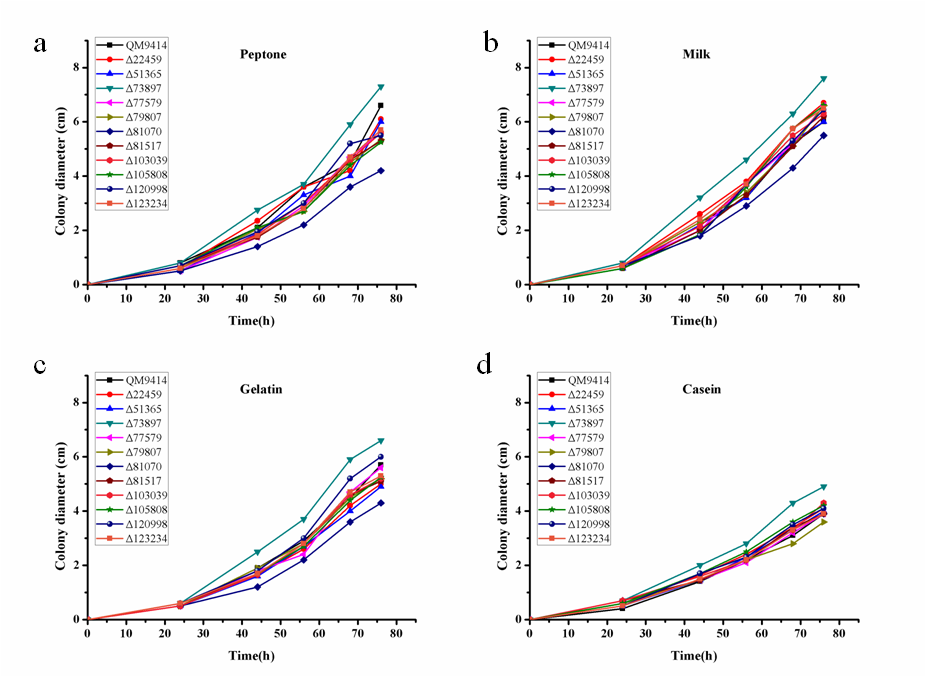
**

**Figure S1** Growth rates of the 11 protease gene-deletion strains and the parental strain QM9414Δ*mus53* (QM9414) on the MM plates containing different nitrogen sources (**a**: peptone; **b**: milk; **c**: gelatin and **d**: casein). The slope of the formula represents the growth rate of the colony. Data are the means of three biological replicates and error bars show the standard deviations.


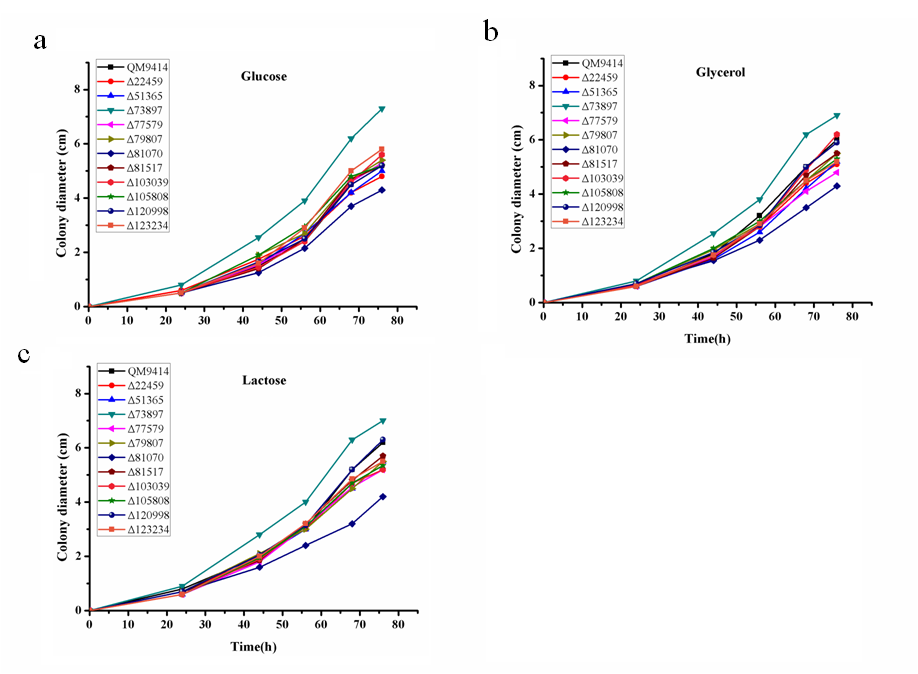


**Figure S2** Growth rates of the 11 protease gene-deletion strains and the parental strain QM9414Δ*mus53* (QM9414) on the MM plates containing different carbon sources (**a**: glucose; **b**: glycerol; **c**: lactose). The slope of the formula represents the growth rate of the colony. Data are represented as the mean of three independent experiments; error bars express the standard deviations.


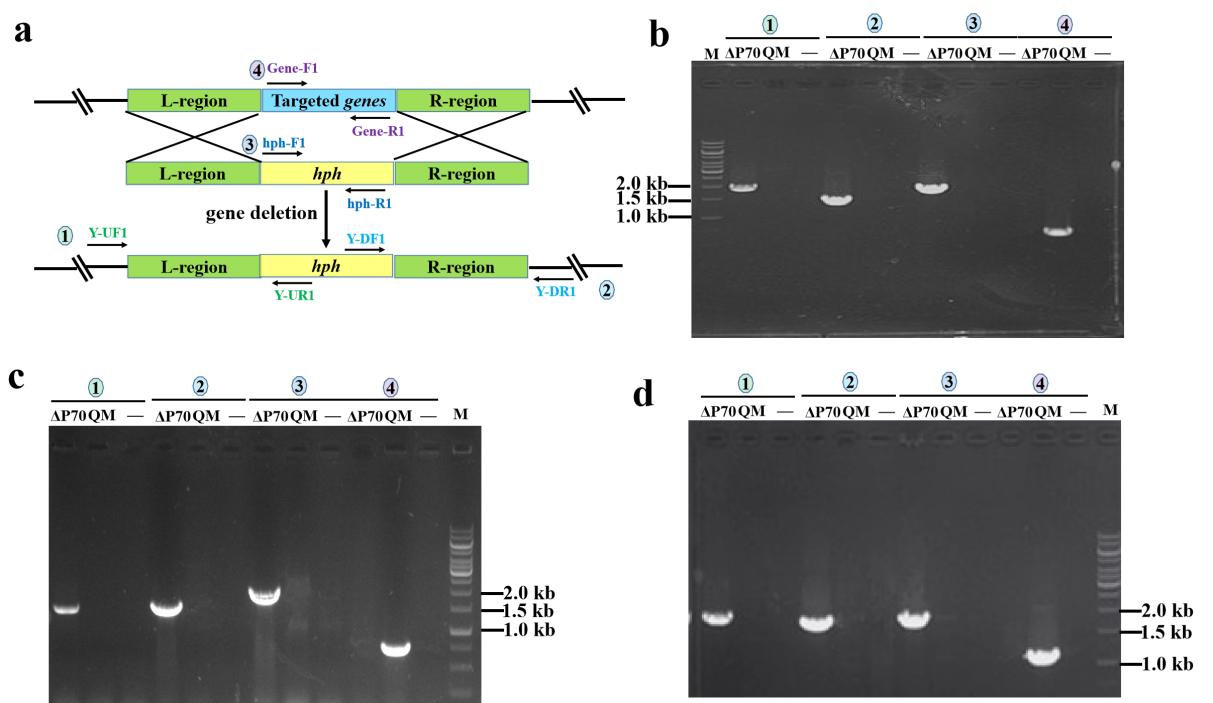


**Figure S3** PCR analysis of the triple protease-gene deletions in *T. reesei* ΔP70. (**a**) Graphical representation of the genomic loci for deletion of the protease genes. (**b**) PCR analysis of the *tre81070* gene deletion in ΔP70. ① The left anchoring region (1.9kb) of the *tre81070* gene deletion spanning from the genomic location to the displaced *hph* gene was amplified with the prime pair① (81070-2081-UF1/Y-hph-121-DF1) in ΔP70 while no product was amplified from the parental strain QM9414Δ*mus53* (QM); ② The right anchoring region (1.5kb) of the *tre81070* gene deletion spanning from the genomic location to the displaced *hph* gene was amplified with the prime pair② (Y-hph-121-DF1/81070-1916-DR1) in ΔP70 while no product was amplified from QM9414Δ*mus53* (QM); ③ The fragment of the *hph* gene (2.0kb) was amplified with the prime pair③ (hph-F/hph-R) in ΔP70 while no product was amplified from QM9414Δ*mus53* (QM); ④ The fragment of the *tre81070* gene (0.9kb) was amplified with the prime pair④ (81070-551-F1/81070-1699-R1) in QM9414Δ*mus53* (QM) while no product was amplified from ΔP70. (**c**) PCR analysis of the *tre120998* gene deletion in ΔP70. ① The left anchoring region (1.7kb) of the *tre120998* gene deletion spanning from the genomic location to the displaced *hph* gene was amplified with the prime pair① (120998-2086-UF1/Y-hph-121-DF1) in ΔP70 while no product was amplified from QM9414Δ*mus53* (QM); ② The right anchoring region (1.7kb) of the *tre120998* gene deletion spanning from the genomic location to the displaced *hph* gene was amplified with the prime pair② (Y-hph-121-DF1/120998-2005-DR1) in ΔP70 while no product was amplified from QM9414Δ*mus53* (QM); ③ The fragment of the *hph* gene (2.0kb) was amplified with the prime pair③ (hph-F/hph-R) in ΔP70 while no product was amplified from QM9414Δ*mus53* (QM); ④ The fragment of the *tre120998* gene (0.8kb) was amplified with the prime pair④ (120998-294-F1/120998-1473-R1) in QM9414Δ*mus53* (QM) while no product was amplified from ΔP70. (**d**) PCR analysis of the *tre123234* gene deletion in ΔP70. ① The left anchoring region (1.9kb) of the *tre123234* gene deletion spanning from the genomic location to the displaced *hph* gene was amplified with the prime pair① (123234-1995-UF1/Y-hph-121-DF1) in ΔP70 while no product was amplified from QM9414Δ*mus53* (QM); ② The right anchoring region (1.8kb) of the *tre123234* gene deletion spanning from the genomic location to the displaced *hph* gene was amplified with the prime pair② (Y-hph-121-DF1/123234-2003-DR1) in ΔP70 while no product was amplified from QM9414Δ*mus53* (QM); ③ The fragment of the *hph* gene (2.0kb) was amplified with the prime pair③ (hph-F/hph-R) in ΔP70 while no product was amplified from QM9414Δ*mus53* (QM); ④ The fragment of the *tre123234* gene (1.1kb) was amplified with the prime pair④ (123234-215-F1/123234-1206-R1) in QM9414Δ*mus53* (QM) while no product was amplified from ΔP70.
